# Persistent effects of swine manure biochar and biogas slurry application on soil nitrogen content and quality of lotus root

**DOI:** 10.3389/fpls.2024.1359911

**Published:** 2024-03-04

**Authors:** Mengyu Zhang, Jiatao Cui, Meng Mi, Zewen Jin, Ming Hung Wong, Shengdao Shan, Lifeng Ping

**Affiliations:** ^1^ Key Laboratory of Recycling and Eco-Treatment of Waste Biomass of Zhejiang Province, Zhejiang University of Science and Technology, Hangzhou, China; ^2^ Consortium on Health, Environment, Education, and Research (CHEER), Department of Science and Environmental Studies, The Education University of Hong Kong, Hong Kong, Hong Kong SAR, China

**Keywords:** swine manure biochar, biogas slurry, nitrogen content, enzyme activity, lotus root

## Abstract

Using swine manure biochar and biogas slurry in agriculture proves to be an effective strategy for soil improvement and fertilization. In this study, a pot trial on the growth of lotus root was conducted to investigate the persistent effects of applying 350°C swine manure biochar (1% and 2%) and biogas slurry (50% and 100%) on soil nitrogen nutrient and lotus root quality. The results showed that compared to chemical fertilizer alone (A0B0), swine manure biochar significantly increased soil nitrogen content after one year of application. The contents of total nitrogen (TN), alkali-hydrolyzed nitrogen (AHN), ammonium nitrogen (NH_4_^+^-N), and nitrate nitrogen ( $NO_{3^{-\;}}-N$ ) increased by 17.96% to 20.73%, 14.05% to 64.71%, 17.76% to 48.68% and 2.22% to 8.47%, respectively, during the rooting period. When swine manure biochar was present, the application of biogas slurry further elevated soil nitrogen content. The co-application of swine manure biochar and biogas slurry significantly increased soil nitrogen content, and the 100% nitrogen replacement with biogas slurry combined with 2% swine manure biochar (A2B2) treatment exhibited the most significant enhancement effect during whole plant growth periods. Soil enzyme activities, including soil protease (NPT), leucine aminopeptidase (LAP), b-glucosidase (β-GC) and dehydrogenase (DHA), showed a tendency to increase and then decrease with the prolongation of lotus root fertility period, reaching the maximum value during the rooting period. Compared to A0B0, the treatment with 2% swine manure biochar had the most significant effect on enzyme activities and increased the lotus root’s protein, soluble sugar, and starch contents. Nitrate content decreased with the application of 2% swine manure biochar as the amount of biogas slurry increased. In conclusion, swine manure biochar effectively improved soil nitrogen content, enzyme activity, and lotus root quality. Even after one year of application, 2% swine manure biochar had the best enhancement effect.

## Graphical Abstract

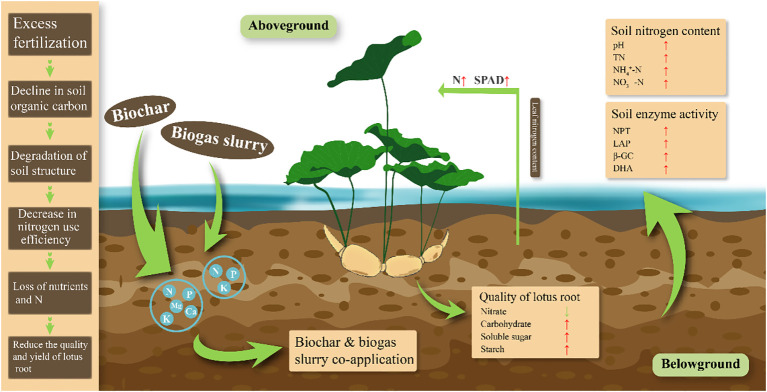

## Highlights

- Swine manure biochar still positively affected soil nitrogen content, enzyme activity, and lotus root quality after one year of application.
- The enhancement of soil nitrogen content, enzyme activity, and lotus root quality was observed as co-application of biochar and biogas slurry > swine manure biochar alone > biogas slurry alone.
- The A2B2 treatment was the most significant enhancement effect of soil improvement and lotus root quality during whole fertility periods of lotus root.

## 1 Introduction

Aquatic vegetables are characteristic vegetables in China. Lotus root (*Nelumbo nucifera Gaertn*) is one of the important types of aquatic vegetables, which contains about 17.64% starch, 3.67% total dietary fiber, 2.56% protein (Yi et al., 2016). Additionally, lotus root contains alkaloids, flavonoids, polyphenols and other bioactive compounds (Zhu, 2017). Its cultivation scale and economic benefits rank prominently among the top of aquatic vegetables. Lotus root is rich in nutrients and has physiological effects of scavenging free radicals, antioxidant and hypoglycemic, therefore having good food and medicinal values (Ding et al., 2023).Lotus root is a crop with a long growth and development cycle. The fertilizer effect period of traditional fertilizer is short, and one-time basal application cannot meet the nutrients required for the whole growth and development period of lotus root. It is necessary to apply fertilizer in different periods to ensure the yield of lotus root (Long, 2013). In the cultivation process of lotus root, in addition to the application of sufficient base fertilizer, the growth period should also be timely staged topdressing 2 to 3 times. Usually, urea 15 kg/mu is applied at the leaf period; urea 30 kg/mu and compound fertilizer 50 kg/mu were applied at the leaf sealing period; urea 30 kg/mu and compound fertilizer 25 kg/mu were applied at the rooting period. The escalating demand for high-quality-lotus roots has led to some farmers indiscriminately use chemical nitrogen fertilizers during cultivation. Excessive application of nitrogen fertilizer will not only reduce the utilization rate of nitrogen fertilizer, but also cause the phenomenon of late maturity of crops, reduce the yield of crops and deteriorate the quality, which will lead to serious environmental pollution and increase the planting cost (Wang and Song, 2020; Wu et al., 2020). Therefore, improving the fertilization technology to create a good growing environment for lotus roots is the key to achieving the yield increase.

Biochar is a carbon-rich and insoluble solid material obtained by high-temperature pyrolysis of agricultural and forestry wastes under anaerobic or anoxic conditions (Premalatha et al., 2023). It primarily comprises carbon (C), hydrogen (H), oxygen (O), nitrogen (N), and other essential plant nutrients such as phosphorus (P), potassium (K), calcium (Ca), and magnesium (Mg) (Danesh et al., 2023). With its developed pore structure, large specific surface area, abundant functional groups, and high stability, biochar is widely used in soil improvement, crop yield increase, and environmental ecological restoration (Rombel et al., 2022). Biogas slurry is the product of anaerobic fermentation of livestock manure, straw, etc. and it is not only rich in N, P, K, and other essential nutrients for plant growth but also rich in amino acids, minerals, humic acids, and vitamins, etc (Jiang et al., 2023). Appropriate application of biogas slurry can partially replace chemical fertilizers and improve crop quality (Lu et al., 2021), which solves the problem of difficulties in livestock and poultry manure treatments (Cui et al., 2021). According to the statistical estimation, China’s annual production of livestock and poultry manure is about 3.8 billion tons, and a series of environmental pollution problems that have to deal with (Liang et al., 2018). The effective utilization of pyrolysis carbonization technology for biochar preparation (Kong et al., 2015) and anaerobic fermentation for biogas slurry production (Shi et al., 2021) is a crucial solution for livestock waste reutilization. Compared to a single application, the co-application of biochar and biogas slurry exhibits positive effects on soil organic matter, microbial carbon and nitrogen content (Zheng et al., 2020b), enzyme activity (Feng et al., 2021), microbial diversity, and crop yield (Zheng et al., 2023). Therefore, this co-application can effectively improve and fertilize the soil and reduce the amount of chemical fertilizers.

Nitrogen is one of the most important nutrients in the growth and development of lotus roots. In the process of lotus root cultivation, the excessive application of chemical fertilizer causes inorganic nitrogen to accumulate in the soil, leading to nitrogen loss and ecological problems through leaching, volatilization, and denitrification (Lehmann et al., 2003; Khan et al., 2021). Biochar and biogas slurry are widely used to improve agricultural soil, which can improve soil properties and affect the migration of soil nutrients (Guo et al., 2014; Liang et al., 2023). At present, the research on the application of biochar and biogas slurry is mainly focused on the improvement of dryland soil properties (Liao et al., 2022; Zhang et al., 2022; Ren et al., 2023), the effect on the properties of flooded soil is rarely investigated (Wang et al., 2023). This study focused on exploring the persistent effects of biochar and biogas slurry co-application on soil nitrogen nutrients and lotus root growth characteristics. Specifically, we investigated the intrinsic effects of swine manure biochar application on soil nitrogen nutrients and lotus root quality after one year of application. The result will contribute to the theoretical foundation and technical support for soil improvement in lotus root fields.

## 2 Materials and methods

### 2.1 Materials for testing

The test soil was collected from the campus base (30°13′N, 120°01′E) of Zhejiang University of Science and Technology, Hangzhou, Zhejiang Province. The soil was classified as red-yellow loam according to the World Reference Base (WRB), with the following basic physical and chemical properties: pH value 5.15, organic carbon 5.23 g·kg^-1^, total nitrogen 0.568 g·kg^-1^, ammonium nitrogen 0.801 mg·kg^-1^, nitrate nitrogen 3.57 mg·kg^-1^, total phosphorus 0.146 g·kg^-1^, total potassium 12.2 g·kg^-1^, alkali-hydrolyzed nitrogen 83.7 mg·kg^-1^, quick-acting phosphorus 1.25 mg·kg^-1^, quick-acting potassium 66 mg·kg^-1^.

The test crop was lotus root (*Nelumbo nucifera Gaertn*), the variety was E-Lian No.6, which was purchased from Yuebo Aquatic Plant Co. Lotus roots were planted in 200 L ceramic vats with an inner diameter of 30 cm at the mouth, an inner diameter of 22 cm at the bottom and a height of 61 cm, and the soil was 85 kg per vat.

The chemical fertilizers used in the experiment included nitrogen fertilizer (CH_4_N_2_O), phosphorus fertilizer (P_2_O_5_) and potash fertilizer (K_2_O), which were purchased from the Nongjia Hospital.

The raw material of biochar used in the experiment was swine manure from Shunkang animal husbandry in Kaihua, Zhejiang Province. The swine manure biochar was prepared by anaerobic carbonization at 350°C by Jinhua Jinguo Company, Zhejiang Province. The basic physical and chemical properties of swine manure biochar were as follows: pH value 7.56, carbon 10.51%, hydrogen 0.97%, nitrogen 0.35%, sulfur 0.31%, ash 16.95%, total phosphorus 15.56 g·kg^-1^, total potassium 14.91 g·kg^-1^.

The biogas slurry used in the experiment was the biogas slurry concentrate prepared by anaerobic fermentation of Shunkang animal husbandry pig farm wastewater in Kaihua, Zhejiang Province. Its basic physical and chemical properties were as follows: pH value 8.66, total nitrogen 33.0 g·L^-1^, ammonia 23.68 g·L^-1^, total phosphorus 13.2 mg·L^-1^, total potassium 5.54 g·L^-1^.

### 2.2 Test methods

The experiment was carried out in the solar greenhouse at the campus base of Zhejiang University of Science and Technology in Hangzhou, Zhejiang Province in, 2022, and the positioning experiment began in April, 2021. In April, 2021, swine manure biochar was applied to the ceramic vats at one time, and water was added after mixing with the soil evenly. The initial water depth was 5 cm, and the positions of each vat were randomly arranged. On April 24,2022, three lotus seedlings were placed in each vat, and water was added to make the initial water depth 7cm. The experiment was conducted in a complete interaction design with swine manure biochar and biogas slurry: three levels of swine manure biochar were set at 0%, 1%, and 2% (mass fraction) and three application amounts of biogas slurry were set at 0%, 50% and 100% (nitrogen substitution), respectively. The experiment consisted of 10 treatments with 3 replicates, and the specific treatments were as follows: (1) control (CK), (2) pure fertilizer (A0B0), (3) 350 °C swine manure biochar 1% (A0B1), (4) 350 °C swine manure biochar 2% (A0B2), (5) 50% nitrogen replacement of biogas slurry (A1B0), (6) 50% nitrogen replacement of biogas slurry + 350 °C swine manure biochar 1% (A1B1), (7) 50% nitrogen replacement of biogas slurry + 350 °C swine manure biochar 2% (A1B2), (8) 100% nitrogen replacement of biogas slurry (A2B0), (9) 100% nitrogen replacement of biogas slurry + 350 °C swine manure biochar 1% (A2B1), (10) 100% nitrogen replacement of biogas slurry + 350 °C swine manure biochar 2% (A2B2). The specific treatment is shown in **Table 1**. CK was without swine manure biochar, biogas slurry, or chemical fertilizer.

**Table 1:** Experimental design scheme.

Treatment	Biogas slurry application	Whether chemical fertilizers were applied	Biochar application	Types of biochar
CK	/	No	/	/
A0B0	/	Yes	/	/
A0B1	0%	Yes	1%	350°C swine manure biochar
A0B2	0%	Yes	2%	350°C swine manure biochar
A1B0	50%	Yes	0%	/
A1B1	50%	Yes	1%	350°C swine manure biochar
A1B2	50%	Yes	2%	350°C swine manure biochar
A2B0	100%	Yes	0%	/
A2B1	100%	Yes	1%	350°C swine manure biochar
A2B2	100%	Yes	2%	350°C swine manure biochar

0 kg of 0% swine manure biochar, 0.85 kg of 1% swine manure biochar, 1.70 kg of 2% swine manure biochar. 0 L for 0% biogas slurry application, 4.2 L for 50% biogas slurry application and 8.4 L for 100% biogas slurry application.

The chemical fertilizer was applied twice in the flowering and fruiting period of lotus root, the amount of each application was half of the total amount. The biogas slurry was applied to lotus root at seedling, leaves-forming, flowering and fruiting, and rooting periods, respectively. The fertilizer application rates for the treatments without biogas slurry were as follows: nitrogen fertilizer (CH_4_N_2_O) 13.3 g/vat, phosphorus fertilizer (P_2_O_5_) 5.33 g/vat and potash fertilizer (K_2_O) 10.66 g/vat. The fertilizer and biogas slurry application rates for the treatments applying biogas slurry were as follows: fertilizer application was calculated as urea (CH_4_N_2_O) 28.9 g/vat, nitrogen content of biogas slurry concentrate was 1.57 g·L^-1^, biogas slurry watering, 1500 mL was 100% nitrogen replacement and biogas slurry watering 750 mL was 50% nitrogen replacement. No nitrogen fertilizer was applied to 100% nitrogen replacement, and phosphate and potash fertilizers were applied normally; the nitrogen fertilizer applied to 50% nitrogen replacement was halved, and phosphate and potash fertilizers were applied normally.

### 2.3 Sample collection and measurement

#### 2.3.1 Soil sample collection and determination

Soil samples were collected once during the seedling, flowering and fruiting, rooting, and dormant periods of lotus roots, respectively. Soil physical and chemical indexes were determined by conventional methods (Bao, 2000): pH value was determined by a pH meter; the organic carbon was determined by the potassium dichromate volumetric method with external heating; the total nitrogen was digested by the concentration of sulfuric acid-mixed catalyst and determined by the flow analyzer; the alkali-hydrolyzed nitrogen was determined by alkali solution diffusion method; the ammonium nitrogen and nitrate nitrogen were extracted by potassium chloride, determined by the flow analyzer; the Suming Technology kit determined soil protease, leucine aminopeptidase, β-glucosidase and dehydrogenase.

#### 2.3.2 Plant sample collection and determination

The leaves were collected once at the early, middle and end period of lotus root formation, rinsed with distilled water, placed in an oven, deactivated at 105 °C for 0.5 h, dried to constant weight at 75 °C, crushed by a grinder, and sieved for later use. The lotus root was collected during the dormant period. After collection, it was washed and dried. One part was stored in the refrigerator at -80 °C, and the other part was dried in the oven (ibid.) to constant weight, crushed and sifted for use. Plants physical and chemical indexes were determined by conventional methods (Bao, 2000): the nitrogen contents of leaves and lotus roots were determined by H_2_SO_4_-H_2_O_2_ digestion pretreatment and determined by the flow analyzer; leaves chlorophyll contents were determined by hand-held chlorophyll meter; the Suming Technology kit determined lotus root nitrate, protein, soluble sugar and starch.

### 2.4 Data analysis

Microsoft Excel, 2016, Origin, 2022 and SPSS 20.0 software were used to analyze the experimental data for analysis of variance, multiple comparisons of means and correlation, with the level of significance set at P = 0.05.

## 3 Results

### 3.1 Effects of biochar and biogas slurry application on soil nitrogen content

#### 3.1.1 Effects of biochar and biogas slurry application on soil pH value

**Figure 1:**
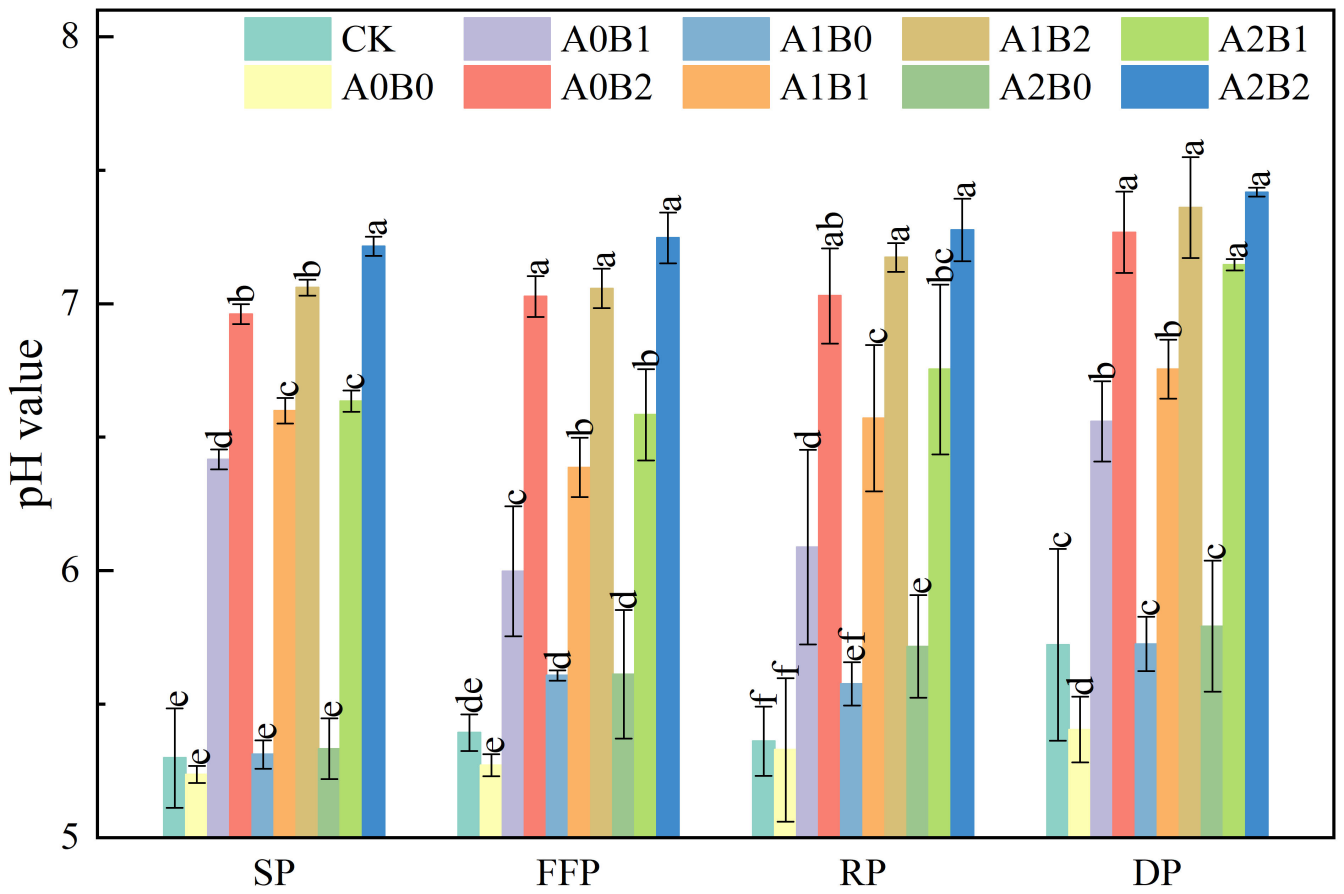
Effects of swine manure biochar and biogas slurry application on soil pH value. Data over bars marked by different letters are significantly different at p < 0.05. Each value represents the mean of three replicates ± SE. SP, seedling period; FFP, flowering and fruiting period; RP, rooting period; DP, dormant period.

The effects of swine manure biochar and biogas slurry application on soil pH value are shown in **Figure 1**. Significant variations in soil pH values were observed among different treatments. Following one year of swine manure biochar application, soil pH values were consistently maintained within the range of 6.00 to 7.27. Compared with the control (CK) and the use of chemical fertilizer alone (A0B0), the soil pH values of swine manure biochar treatment increased by 11.19% to 31.37% and 13.79% to 34.46%, respectively. Soil pH values enhancement were the most obvious at 2% of swine manure biochar application, with 1.67 to 1.86 units of enhancement compared to CK and A0B0. Compared with CK, soil pH values were increased by 3.96% to 4.04% after applying biogas slurry, but there was no significant difference between A1B0 and A2B0 (p > 0.05).

However, when biogas slurry was reapplied in the presence of swine manure biochar, a significant increase in soil pH values of 18.40% to 36.17% were noted compared to CK (P < 0.05). The highest soil pH value was recorded under the A2B2 treatment, which was 1.92 units higher than CK. Lotus root is suitable to grow in the environment with a pH range of 5.60 to 7.50. The influence of swine manure biochar on soil pH proved to be superior to that of biogas slurry, which indicated that the increase in soil pH value could still be maintained continuously after one year of swine manure biochar application. The co-application of swine manure biochar and biogas slurry consistently maintained soil pH values of 6.39 to 7.42. Notably, A2B2 and A1B2 raised soil pH values to approximately 7.00, which were more suitable for the growth of lotus roots.

#### 3.1.2 Effects of biochar and biogas slurry application on soil total nitrogen content

**Figure 2:**
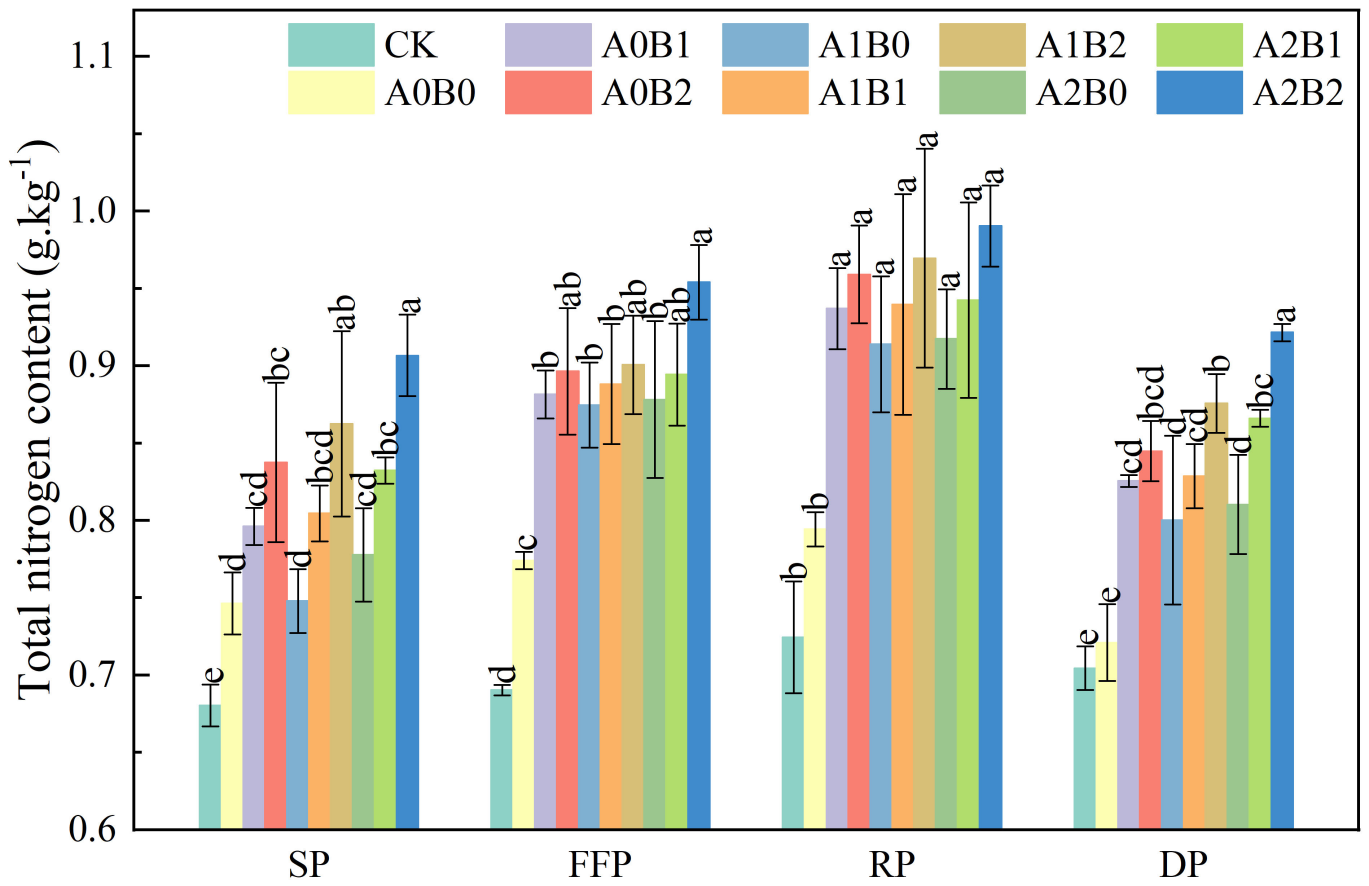
Effects of swine manure biochar and biogas slurry application on soil total nitrogen content. Data over bars marked by different letters are significantly different at p < 0.05. Each value represents the mean of three replicates ± SE. SP, seedling period; FFP, flowering and fruiting period; RP, rooting period; DP, dormant period.

The effects of swine manure biochar and biogas slurry application on soil total nitrogen (TN) content are shown in **Figure 2**. Soil TN content tended to increase and decrease during the fertility period of lotus root, reaching the maximum value at the rooting period. Swine manure biochar still elevated soil TN content after one year of application. Compared with A0B0, the soil TN content increased by 13.87% to 15.79% and 17.96% to 20.73% during the lotus root’s flowering and fruiting, and rooting periods, respectively. Soil TN content reached a maximum value of 0.959 g·kg^-1^ at 2% swine manure biochar application during the rooting period. Compared with A0B0, biogas slurry application elevated soil TN content by 12.98% to 13.44% and 15.06% to 15.48% at flowering and fruiting, and rooting periods, respectively. Although biogas slurry application elevated soil TN content, the difference between biogas slurry treatments was insignificant (P > 0.05). Compared with CK and A0B0, the reapplication of biogas slurry in the presence of swine manure biochar significantly elevated soil TN content (P < 0.05), with an increase of 29.72% to 36.72% and 18.29% to 24.67%, respectively, during the rooting period. Soil TN content of 0.990 g·kg^-1^ was highest under A2B2 treatment. Compared with CK and A0B0, soil TN content was elevated in all treatments during the rooting period (18 months of swine manure biochar application). All of them reached the maximum value (0.914 ~ 0.990 g·kg^-1^). Still, the treatments’ differences were insignificant (P > 0.05).

In summary, soil TN content remained high after one year of 2% swine manure biochar application. It may be that swine manure biochar itself contains nitrogen. When applied to the soil, it will enhance soil nitrogen content. In addition, swine manure biochar includes a large specific surface area and oxygen-containing functional groups. Still, it exhibits good adsorption and fixation nitrogen capacity after one year of application, increasing soil TN content. Soil TN content was highest under the A2B2 treatment. It is possible that the surface functional group adsorption of swine manure biochar and the slow release of nitrogen from biogas slurry in the presence of swine manure biochar result in a significant increase of soil TN content (Chan et al., 2008; Kocatürk-Schumacher et al., 2019).

#### 3.1.3 Effects of biochar and biogas slurry application on soil alkali-hydrolyzed nitrogen content

**Figure 3:**
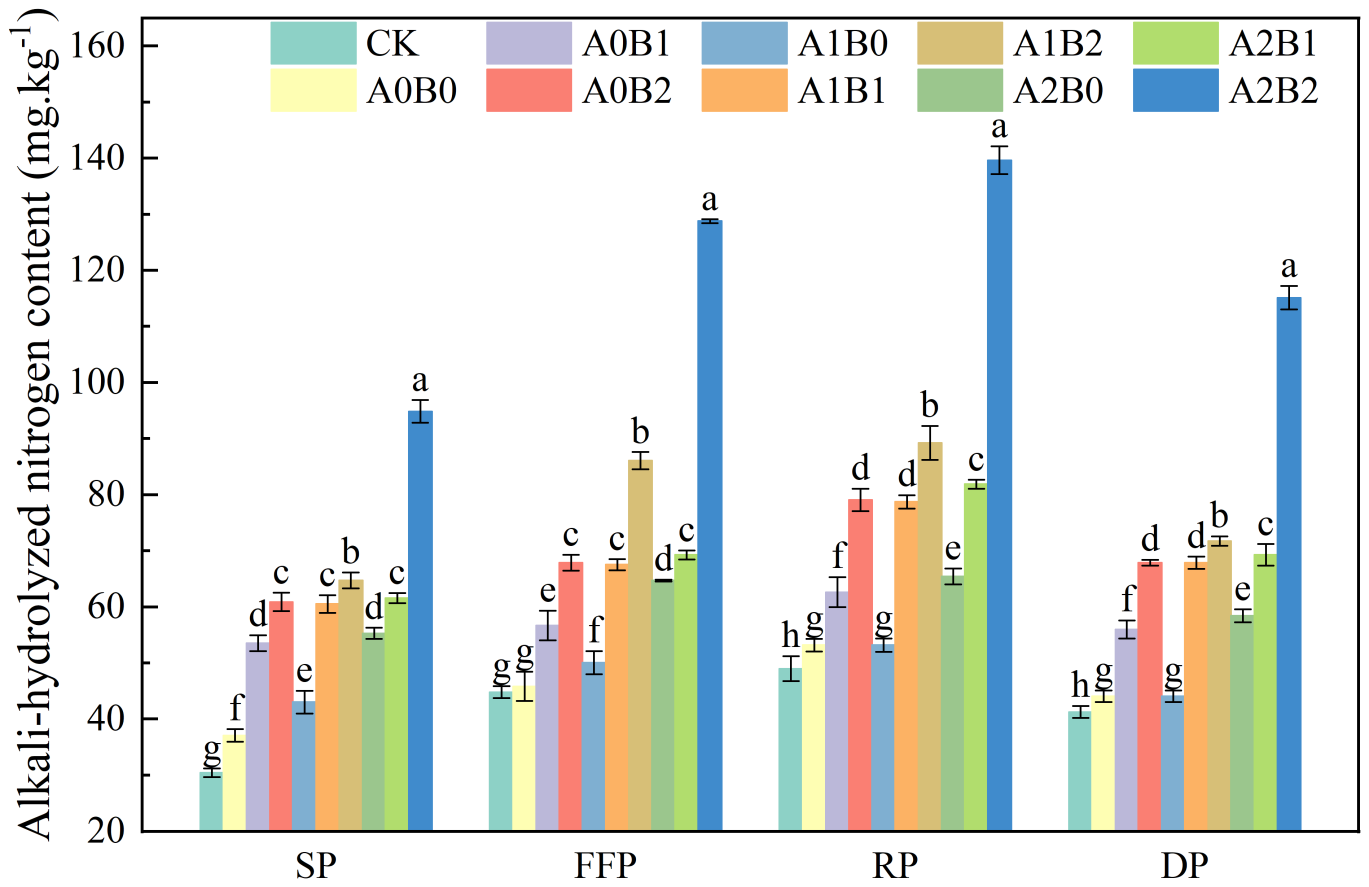
Effects of swine manure biochar and biogas slurry application on soil alkali-hydrolyzed nitrogen content. Data over bars marked by different letters are significantly different at p < 0.05. Each value represents the mean of three replicates ± SE. SP, seedling period; FFP, flowering and fruiting period; RP, rooting period; DP, dormant period.

The effects of swine manure biochar and biogas slurry application on soil alkali-hydrolyzed nitrogen (AHN) content are shown in **Figure 3**. After one year of application of swine manure biochar, it still showed a significant increase in soil AHN content. Compared with A0B0, the soil AHN content of swine manure biochar treatment increased by 44.34% to 64.15%, 23.66% to 48.09%, 17.76% to 48.68% and 26.98% to 53.97% in seedling, flowering and fruiting, rooting and dormant periods, respectively. Soil AHN content was as high as 79.10 mg·kg^-1^ at 2% swine manure biochar application during the rooting period. Biogas slurry application also increased soil AHN content, with a 23.03% increase in A2B0 over A0B0 during the rooting period. In the presence of swine manure biochar, biogas slurry application increased soil AHN content more significantly (P < 0.05), with an increase of 48.03% to 162.50% compare to A0B0 during the rooting period. Under A2B2 treatment, the soil AHN content reached a maximum value of 139.65 mg·kg^-1^.

#### 3.1.4 Effects of biochar and biogas slurry application on soil ammonium nitrogen and nitrate nitrogen content

**Figure 4:**
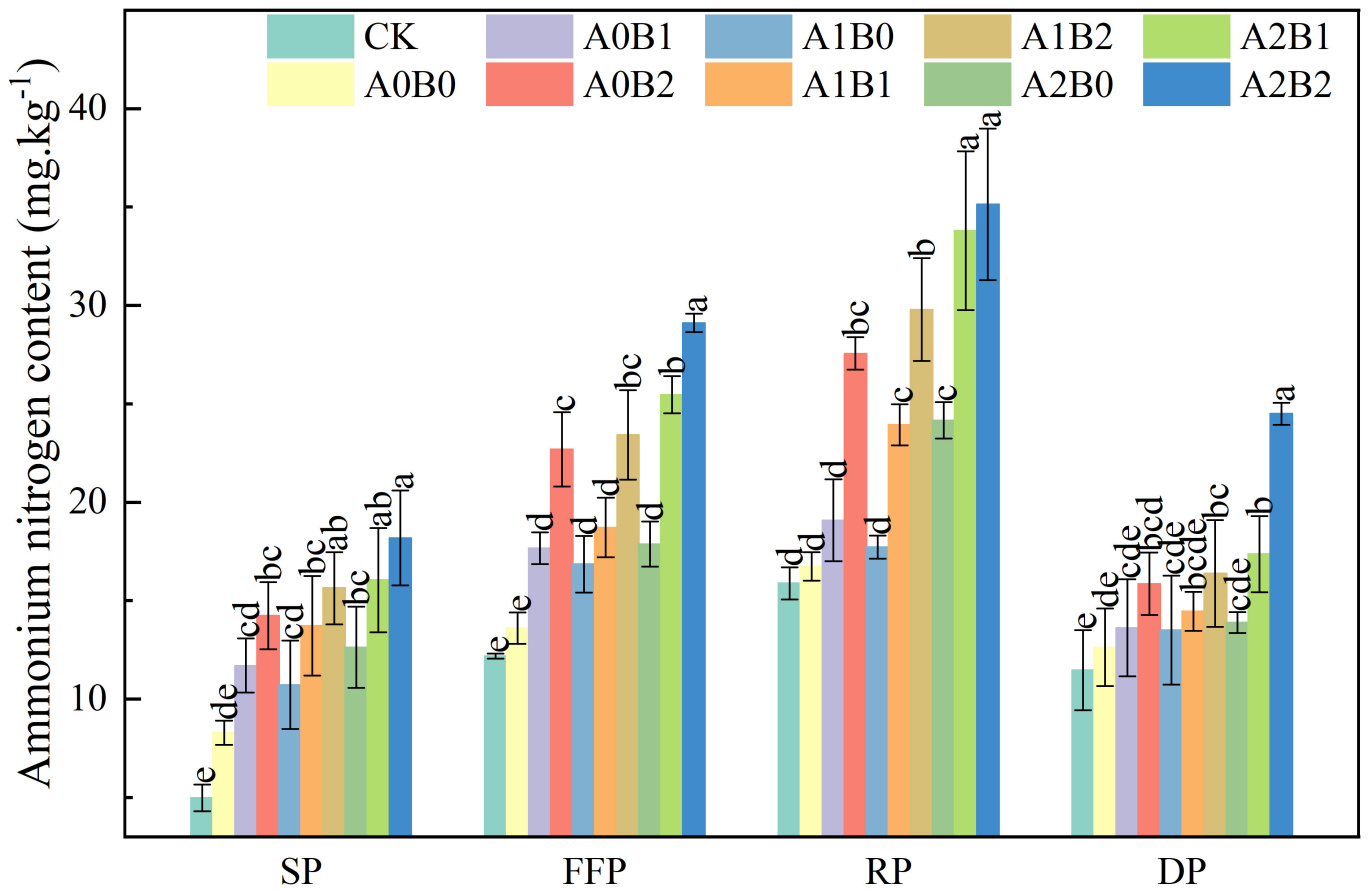
Effects of swine manure biochar and biogas slurry application on soil ammonium nitrogen content. Data over bars marked by different letters are significantly different at p < 0.05. Each value represents the mean of three replicates ± SE. SP, seedling period; FFP, flowering and fruiting period; RP, rooting period; DP, dormant period.

The effects of swine manure biochar and biogas slurry application on soil ammonium nitrogen (NH_4_^+^-N) content are shown in **Figure 4**. Under swine manure biochar treatment, there was a substantial increase in the soil NH_4_^+^-N content, ranging from 41.04% to 71.66%, 29.93% to 66.74%, 14.05% to 64.71% and 7.79% to 25.58% during the seedling, flowering and fruiting, rooting and dormant periods, respectively, compared with A0B0. The highest NH_4_^+^-N content was observed during the rooting period, reaching a peak value of 27.56 mg·kg^-1^ with 2% swine manure biochar application. Compared to A0B0, biogas slurry application also increased soil NH_4_^+^-N content by 29.30% to 52.25% and 5.90% to 44.37% during the seedling and rooting periods, respectively. The co-application of swine manure biochar and biogas slurry significantly enhanced soil NH_4_^+^-N content across all fertility periods. Soil NH_4_^+^-N content in flowering and fruiting, and dormant periods was elevated, but the difference was insignificant between A1B0 and A2B0 (P > 0.05). Soil NH_4_^+^-N content was significantly enhanced under swine manure biochar and biogas slurry co-application during all fertility periods. Under A2B2 treatment, soil NH_4_^+^-N content reached maximum values of 18.18 mg·kg^-1^, 29.11 mg·kg^-1^, 35.14 mg·kg^-1^, and 24.50 mg·kg^-1^ during whole fertility periods, respectively. The soil NH_4_^+^-N content of all treatments reached the maximum value (17.72 ~ 35.14 mg·kg^-1^) during the rooting period.

**Figure 5:**
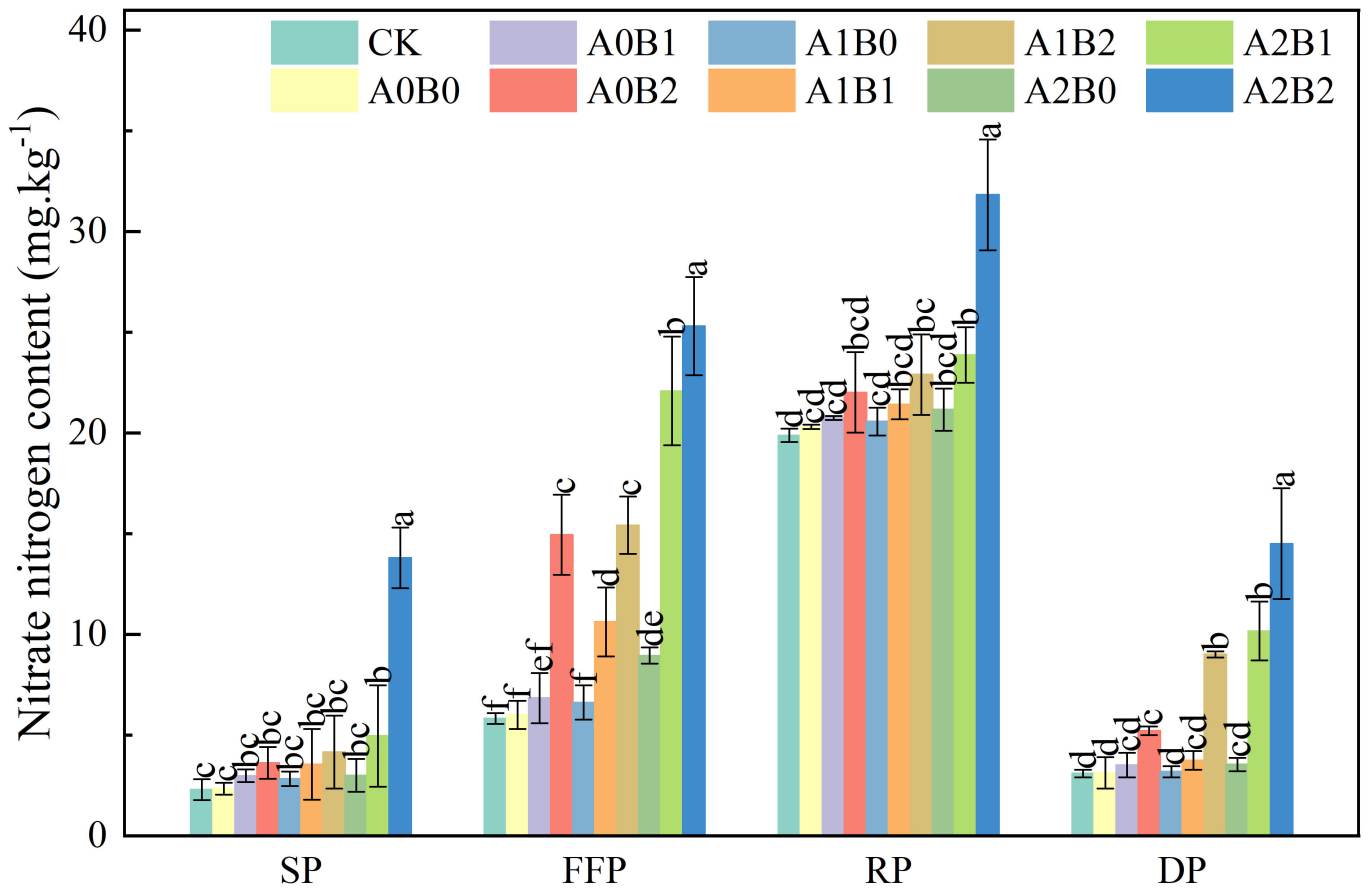
Effects of swine manure biochar and biogas slurry application on soil nitrate nitrogen content. Data over bars marked by different letters are significantly different at p < 0.05. Each value represents the mean of three replicates ± SE. SP, seedling period; FFP, flowering and fruiting period; RP, rooting period; DP, dormant period.

The effects of swine manure biochar and biogas slurry application on soil nitrate nitrogen (*NO*_3^−^_-*N*) content is shown in **Figure 5**. Soil *NO*_3^−^_-*N* content showed a trend of increasing and decreasing with the prolongation of the lotus root fertility period. The soil *NO*_3^−^_-*N* content reached the maximum value during the rooting period. After one year, swine manure biochar continued to promote soil *NO*_3^−^_-*N* content. Compared with A0B0, soil *NO*_3^−^_-*N* content increased by 2.22% to 8.47%. The highest *NO*_3^−^_-*N* content was observed during the rooting period, reaching a peak value of 22.02 mg·kg^-1^ with 2% swine manure biochar application. Compared to A0B0, soil *NO*_3^−^_-*N* content increased by 1.37% to 4.24% under the application of biogas slurry and the highest *NO*_3^−^_-*N* content was observed during the rooting period. Compared to A0B0, co-application of swine manure biochar and biogas slurry significantly increased soil *NO*_3^−^_-*N* content by 5.57% to 56.78% during the rooting period (P < 0.05). Soil *NO*_3^−^_-*N* content reached a maximum value of 31.83 mg·kg^-1^ under A2B2 treatment.

In summary, swine manure biochar exhibited a sustained enhancement effect on soil NH_4_^+^-N and *NO*_3^−^_-*N* content even after one year of application. The most significant enhancement effect was shown under 2% swine manure biochar application. The A2B2 treatment significantly improved soil NH_4_^+^-N and *NO*_3^−^_-*N* content (p < 0.05).

### 3.2 Effects of biochar and biogas slurry application on soil enzyme activity

#### 3.2.1 Effects of biochar and biogas slurry application on soil protease activity

**Figure 6:**
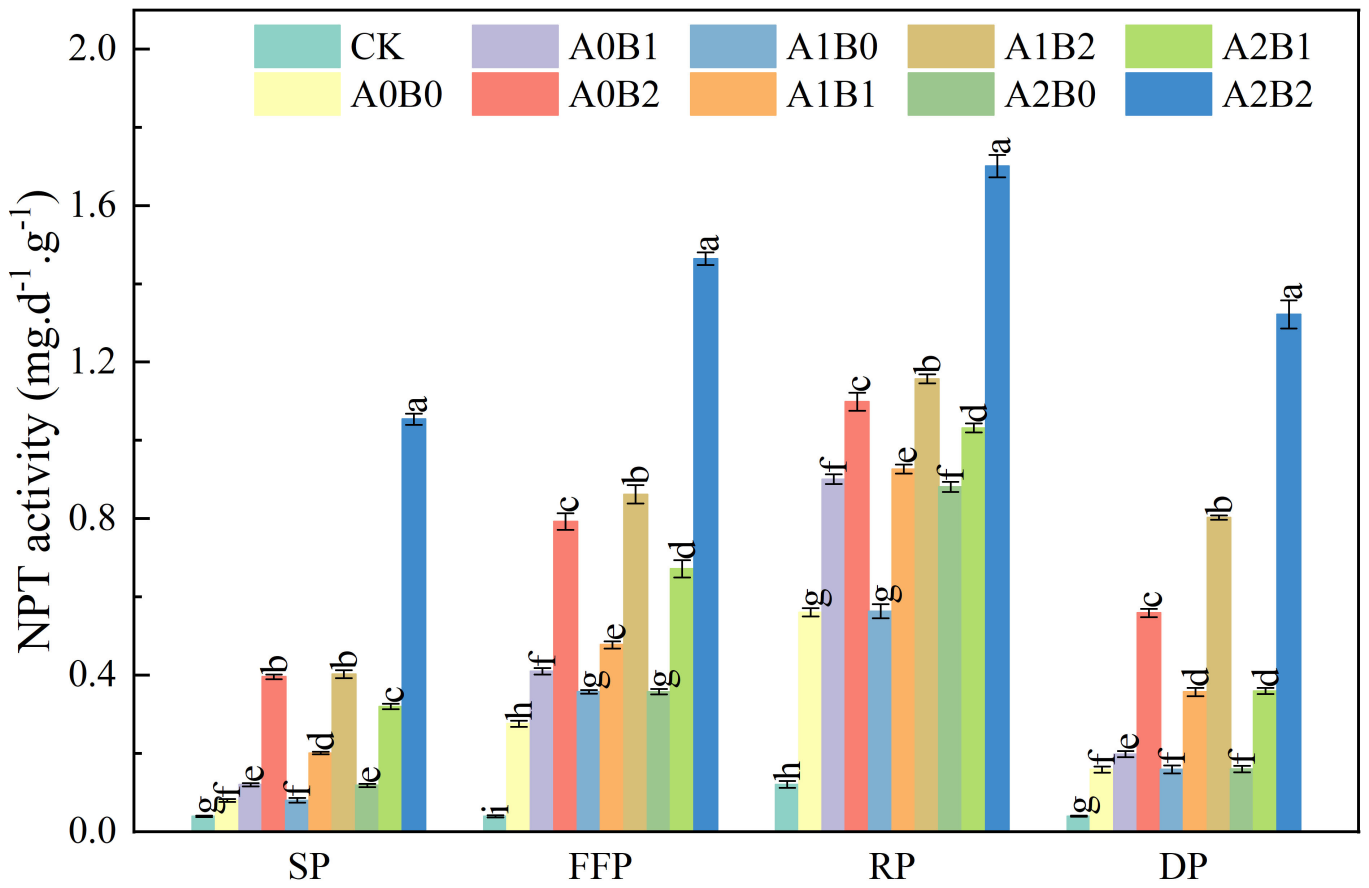
Effects of swine manure biochar and biogas slurry application on soil protease activity. Data over bars marked by different letters are significantly different at p < 0.05. Each value represents the mean of three replicates ± SE. SP, seedling period; FFP, flowering and fruiting period; RP, rooting period; DP, dormant period.

Soil proteases (NPT) play a crucial role in converting amino acids, proteins, and other organic compounds containing protein nitrogen in the soil. The hydrolysis products of these enzymes are one of the major sources of nitrogen for higher plants. The effects of swine manure biochar and biogas slurry application on soil NPT activity are shown in **Figure 6**. With the prolongation of the lotus root fertility period, soil NPT activity showed an increasing and then decreasing trend. The overall soil NPT activity reached the maximum value during the rooting period. Swine manure biochar still significantly increased soil NPT activity after one year of application. The highest soil NPT activity was observed during the rooting period, reaching a peak value of 1.10 mg·d^-1^·g^-1^ with 2% swine manure biochar application. Compared to A0B0, biogas slurry application also increased soil NPT activity, but the difference between A1B0 and A2B0 was not significant (P > 0.05). Co-application of swine manure biochar and biogas slurry significantly elevated soil NPT activity (P < 0.05). Under A2B2 treatment, the soil NPT activity reached a maximum of 1.70 mg·d^-1^·g^-1^ during the rooting period.

#### 3.2.2 Effects of biochar and biogas slurry application on soil leucine aminopeptidase activity

**Figure 7:**
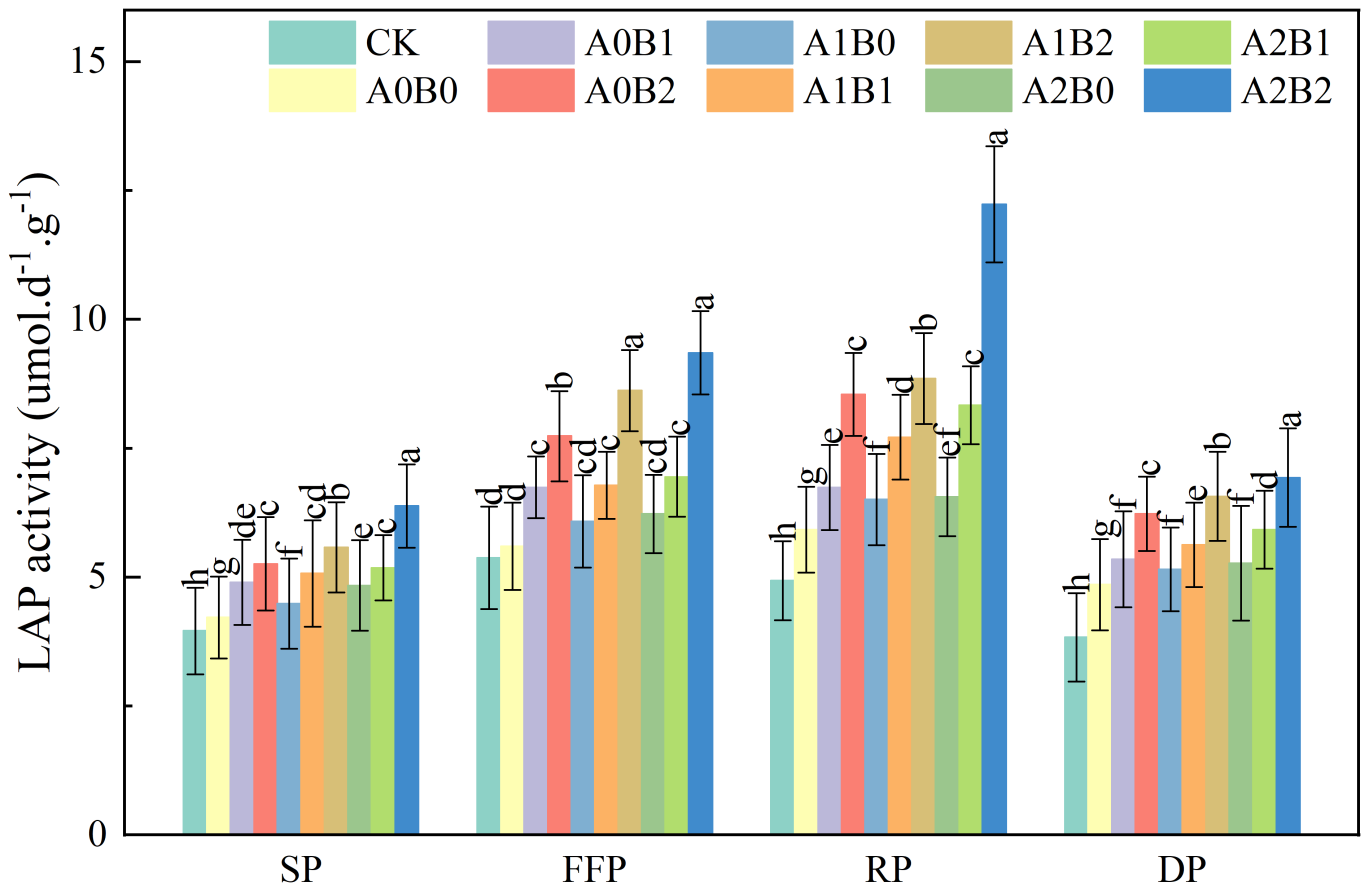
Effects of swine manure biochar and biogas slurry application on soil leucine aminopeptidase activity. Data over bars marked by different letters are significantly different at p < 0.05. Each value represents the mean of three replicates ± SE. SP, seedling period; FFP, flowering and fruiting period; RP, rooting period; DP, dormant period.

Soil leucine aminopeptidase (LAP) is associated with soil nitrogen cycling and acts mainly on the hydrolysis of leucine located at the ends of polypeptides and other hydrophobic amino acids. The effects of swine manure biochar and biogas slurry application on soil LAP activity are shown in **Figure 7**. Compared to A0B0, swine manure biochar continued to increase soil LAP activity after one year of application. The increases of 16.26% to 24.73%, 20.32% to 38.07%, 13.80% to 44.30% and 10.09% to 28.28% were observed during seedling, flowering and fruiting, rooting, and dormant periods, respectively. Applying 2% swine manure biochar resulted in the highest soil LAP activity and reached a maximum value of 8.55 μmol·d^-1^·g^-1^ during the rooting period. Biogas slurry application could also increase soil LAP activity, with an increase of 9.86% to 10.70% compared with A0B0 during the rooting period. However, the difference between A1B0 and A2B0 was insignificant (P > 0.05). The co-application of swine manure biochar and biogas slurry significantly increased soil LAP activity (P < 0.05). Under A2B2 treatment, soil LAP activity reached a maximum of 12.23 μmol·d^-1^·g^-1^ during the rooting period.

#### 3.2.3 Effects of biochar and biogas slurry application on soil β-glucosidase activity

**Figure 8:**
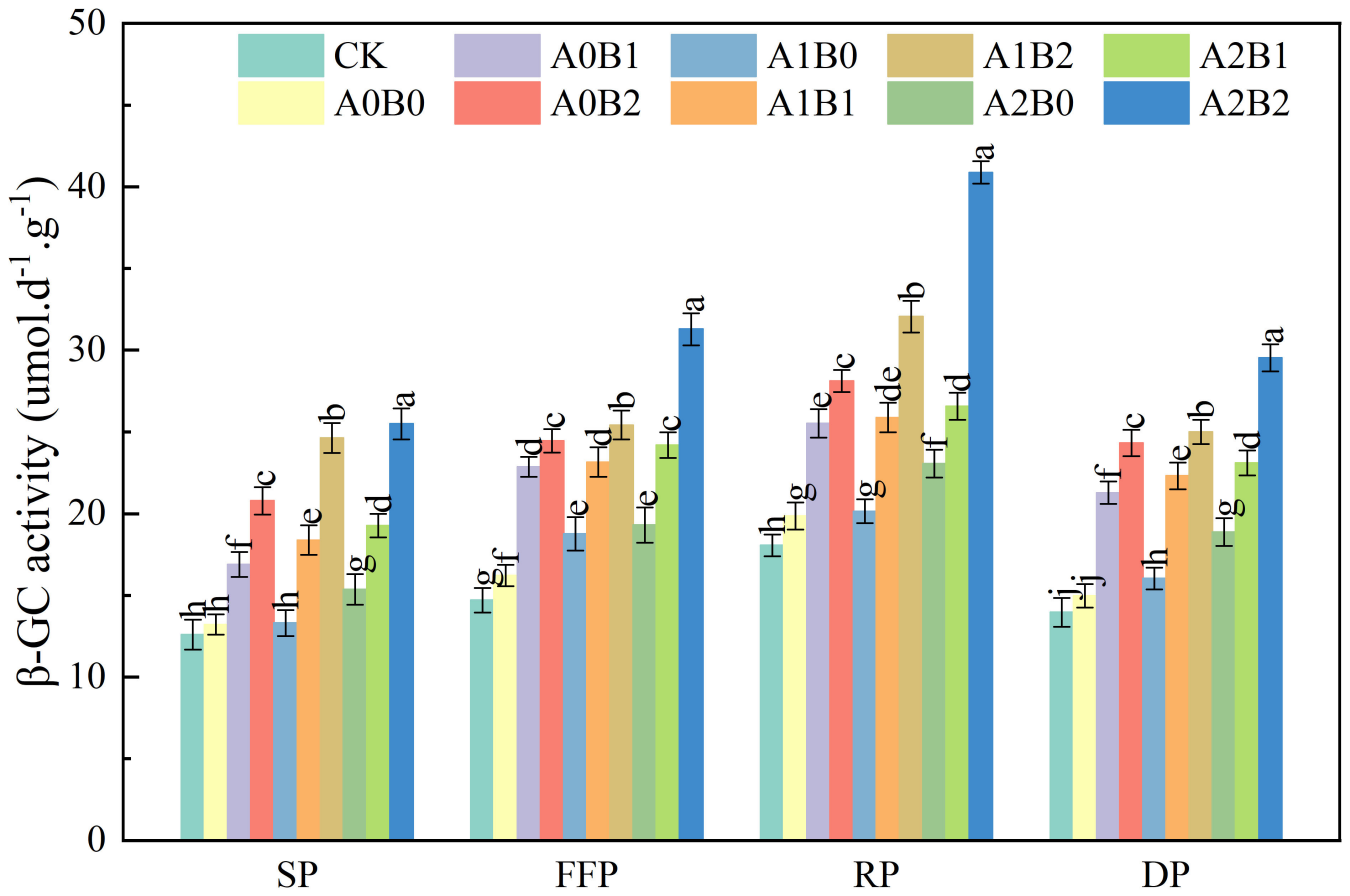
Effects of swine manure biochar and biogas slurry application on soil β-glucosidase activity. Data over bars marked by different letters are significantly different at p < 0.05. Each value represents the mean of three replicates ± SE. SP, seedling period; FFP, flowering and fruiting period; RP, rooting period; DP, dormant period.

Soil β-glucosidase (β-GC) is involved in the mineralization and decomposition of soil organic matter, thus hydrolyzing simpler polysaccharides into glucose monosaccharides. This enzymatic process is closely associated with cellulose degradation, glucose release, and carbon cycling. Soil β-GC is an extremely important microbial energy source. The effects of swine manure biochar and biogas slurry application on soil β-GC activity are shown in **Figure 8**. Compared to A0B0, the application of swine manure biochar significantly increased soil β-GC activity by 27.86% to 57.37%, 41.01% to 50.83%, 28.56% to 41.58% and 42.17% to 62.49% during the seedling, flowering and fruiting, rooting and dormant periods, respectively. Soil β-GC activity under 2% swine manure biochar treatment reached 28.12 μmol·d^-1^·g^-1^ during the rooting period. Compared to A0B0, biogas slurry application also increased soil β-GC activity by 1.43% to 16.13% during the rooting period. Co-application of swine manure biochar and biogas slurry significantly increased soil β-GC activity (P < 0.05). Soil β-GC activity was as high as 40.89 μmol·d^-1^·g^-1^ under A2B2 treatment during the rooting period.

#### 3.2.4 Effects of biochar and biogas slurry application on soil dehydrogenase activity

**Figure 9:**
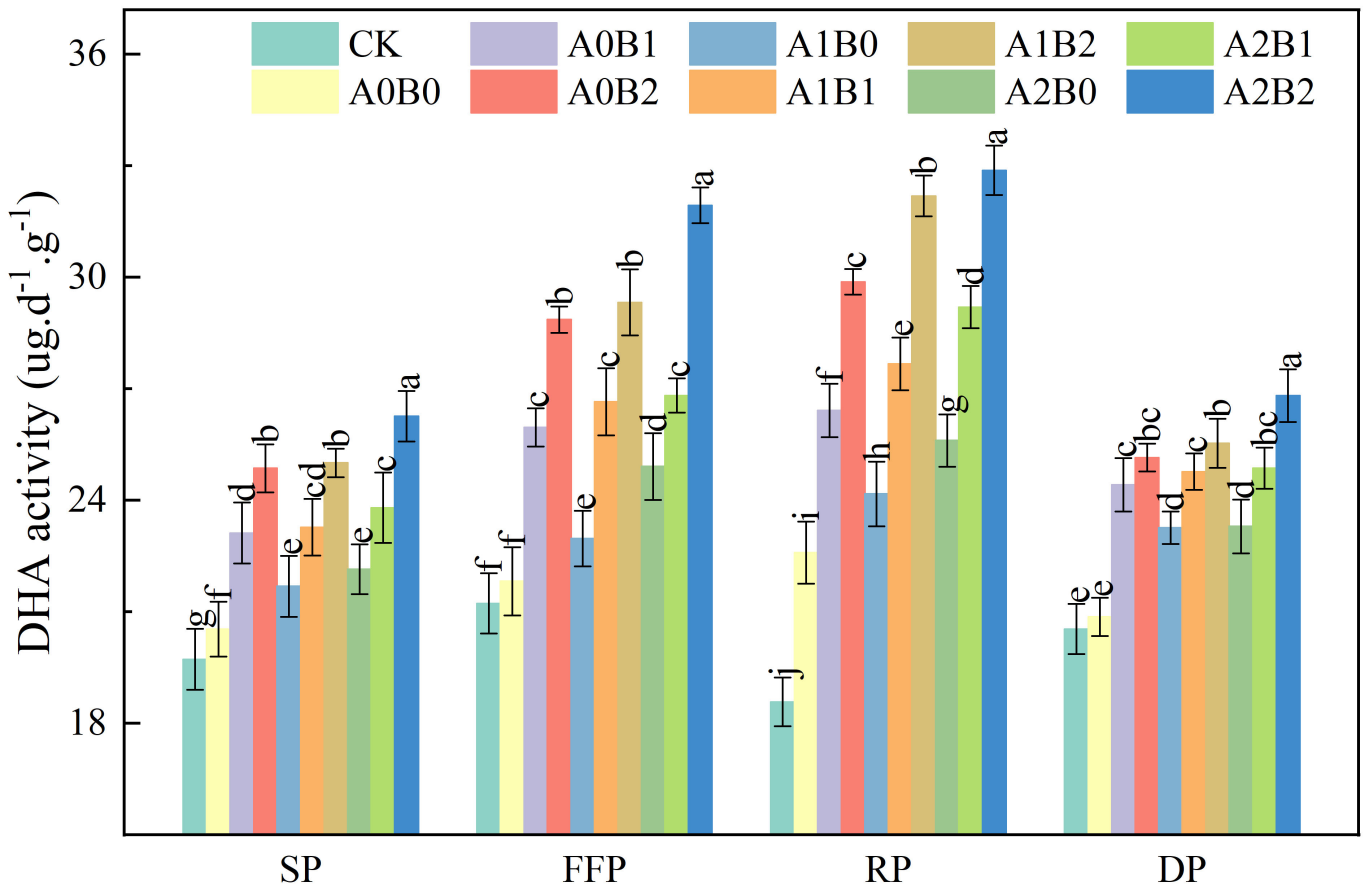
Effects of swine manure biochar and biogas slurry application on soil dehydrogenase activity. Data over bars marked by different letters are significantly different at p < 0.05. Each value represents the mean of three replicates ± SE. SP, seedling period; FFP, flowering and fruiting period; RP, rooting period; DP, dormant period.

Soil dehydrogenase (DHA) catalyzes the redox reactions of sugars, organic acids, amino acids and other substances in the soil. Its activity serves as an indicator of the abundance of active microorganisms in the soil system. Soil DHA is also an important indicator for judging the degradation performance of soil microorganisms. The effects of swine manure biochar and biogas slurry application on soil DHA activity are shown in **Figure 9**. Swine manure biochar continued to increase soil DHA activity after one year of application. Compared with A0B0, soil DHA activity under swine manure biochar treatment increased by 12.56% to 21.05%, 18.98% to 32.25%, 16.93% to 32.25% and 16.98% to 20.51% during the seedling, flowering and fruiting, rooting and dormant periods, respectively. Soil DHA activity reached a maximum value of 29.88 μg·d^-1^·g^-1^ under 2% swine manure biochar application during rooting. The A2B2 treatment recorded the highest soil DHA activity, reaching a maximum of 32.88 μg·d^-1^·g^-1^ during the rooting period. Biogas slurry application also increased soil DHA activity. Compared with A0B0, soil DHA activity increased by 5.59% to 7.82%, 5.26% to 14.16%, 7.01% to 13.35%, and 11.46% to 11.64% under biogas slurry application during the seedling, flowering and fruiting, rooting and dormant periods, respectively. However, the difference between A1B0 and A2B0 (P > 0.05) was insignificant. Co-application of swine manure biochar and biogas slurry significantly increased soil DHA activity (P < 0.05). Soil DHA activity reached a maximum of 32.88 μg·d^-1^·g^-1^ under A2B2 treatment during the rooting period.

### 3.3 Effects of biochar and biogas slurry application on nitrogen content of lotus root

#### 3.3.1 Effects of biochar and biogas slurry application on nitrogen content of leaves and lotus root

**Figure 10:**
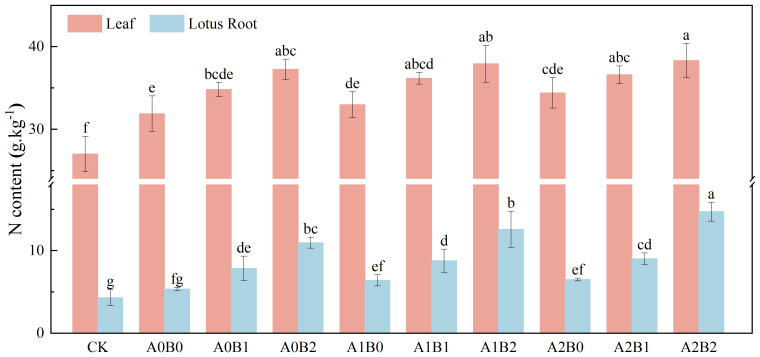
Effects of swine manure biochar and biogas slurry application on nitrogen content of leaves and lotus root. Data over bars marked by different letters are significantly different at p < 0.05. Each value represents the mean of three replicates ± SE.

The effects of swine manure biochar and biogas slurry application on the nitrogen content of leaves and lotus roots are shown in **Figure 10**. The leaves’ nitrogen contents were greater than the corresponding lotus root nitrogen contents under different treatments. Compared to A0B0, the nitrogen content of leaves and lotus root increased by 9.17% to 16.81% and 46.87% to 104.95% after swine manure biochar application. The nitrogen content of leaves and lotus roots reached 37.25 g·kg^-1^ and 10.95 g·kg^-1^ after 2% swine manure biochar application, respectively.

Compared to A0B0, biogas slurry application increased leaves and lotus root nitrogen content by 3.40% to 7.91% and 19.84% to 21.74%, respectively. However, the difference between A1B0 and A2B0 was insignificant (P > 0.05). The co-application of swine manure biochar and biogas slurry significantly increased leaves and lotus root nitrogen content by 13.41% to 20.19% and 64.08% to 175.27%, respectively. The A2B2 treatment showed the highest nitrogen content in leaves (38.33 g·kg^-1^) and lotus root (14.71 g·kg^-1^), respectively. In summary, the co-application of swine manure biochar and biogas slurry significantly increased nitrogen content in leaves and lotus root, and the differences among treatments were significant (p < 0.05). The A2B2 treatment was the most effective in enhancing the nitrogen content of leaves and lotus root.

#### 3.3.2 Effects of biochar and biogas slurry application on leaves chlorophyll

**Figure 11:**
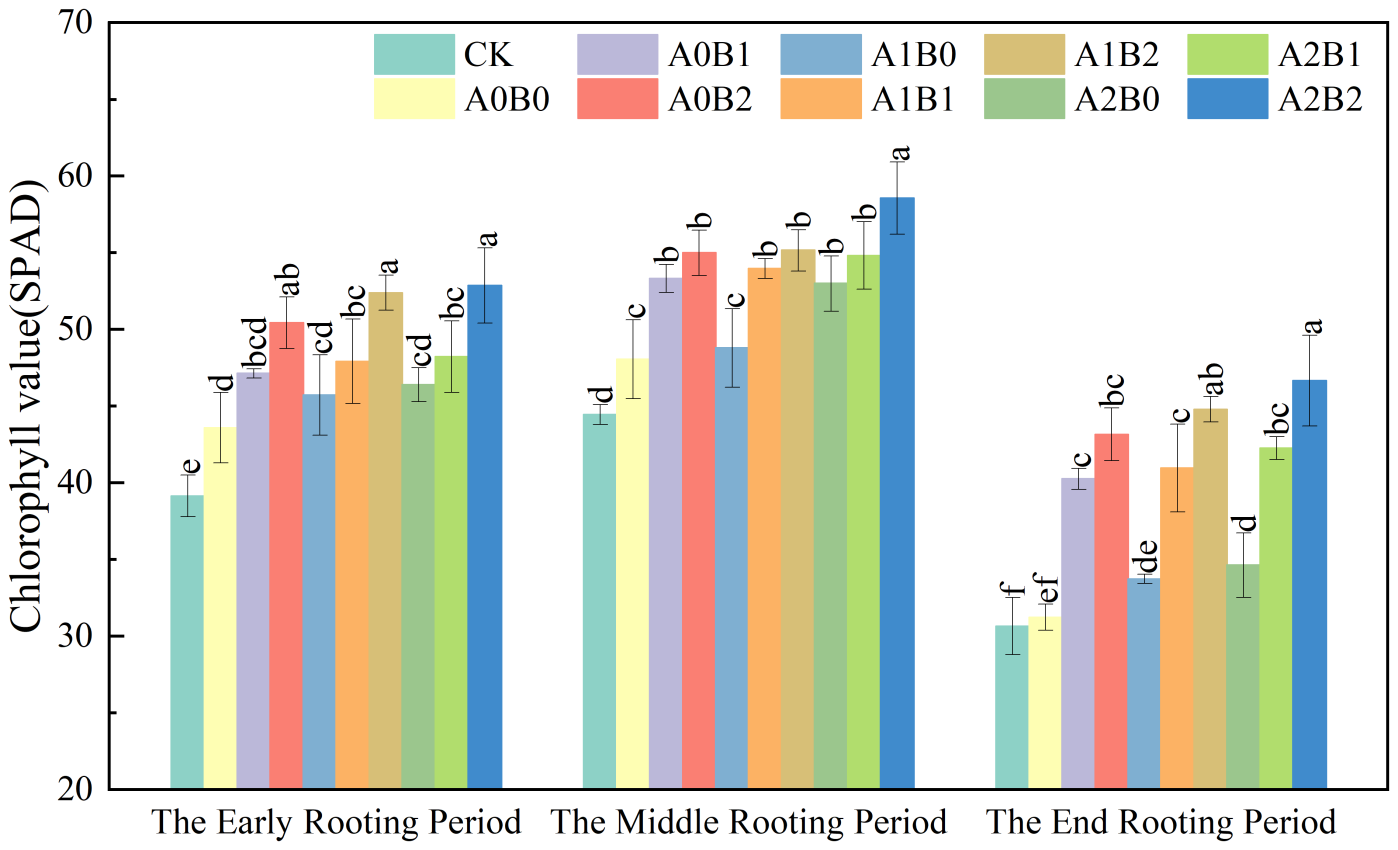
Effects of swine manure biochar and biogas slurry application on leaves chlorophyll value. Data over bars marked by different letters are significantly different at p < 0.05. Each value represents the mean of three replicates ± SE.

Nitrogen absorption, assimilation, and transport in lotus roots can affect the chlorophyll content of leaves. Chlorophyll is the main chemical substance for photosynthesis in plants, and the level of content will directly affect the photosynthetic capacity of the crop and then affect its nutritional status. The effects of swine manure biochar and biogas slurry application on leaves chlorophyll are shown in **Figure 11**. Leaves SPAD values showed a trend of increasing and then decreasing during the rooting period. The values were higher at the middle rooting period than at the early and end rooting period. Compared to A0B0, leaves SPAD values of swine manure biochar treatment increased by 8.10% to 15.67%, 10.96% to 14.42%, and 28.92% to 38.21% at the early, middle, and end rooting periods, respectively. The highest leaves SPAD values (55.0) were recorded at the middle rooting period with a 2% swine manure biochar application.

Compared to A0B0, biogas slurry application increased SPAD values by 4.89% to 6.42%, 1.53% to 10.26%, and 8.00% to 10.89% from the early to the end of rooting. However, the difference between A1B0 and A2B0 was insignificant (P > 0.05). Compared to CK and A0B0, co-application of swine manure biochar and biogas slurry enhanced leaves SPAD values by 21.36% to 31.71% and 12.27% to 21.84% at middle rooting period. The leaves’ SPAD value reached a maximum value (58.6) under A2B2 treatment. In summary, 2% swine manure biochar application continued to have a significant effect on leaves’ SPAD values after one year. The A2B2 treatment had the largest leaf SPAD value during the rooting period, followed by A1B2 and A0B2.

### 3.4 Effects of biochar and biogas slurry application on quality of lotus root

#### 3.4.1 Effects of biochar and biogas slurry application on lotus root quality

The effects of swine manure biochar and biogas slurry application on the lotus root quality are shown in **Table 2**. Compared with A0B0, applying swine manure biochar and biogas slurry significantly increased the protein, soluble sugar, and starch contents of lotus root. The enhancement of protein, soluble sugar, and starch contents of lotus root was observed as co-application of biochar and biogas slurry > swine manure biochar alone > biogas slurry alone. Compared with A0B0, the protein, soluble sugar, and starch contents of lotus roots under swine manure biochar treatments were increased by 42.68% to 66.00%, 15.20% to 58.89% and 7.88% to 19.18%, respectively, maximum values were attained at 2% swine manure biochar application. Biogas application increased the contents of protein, soluble sugar and starch of lotus roots by 16.08% to 17.71%, 0.57% to 14.11% and 6.17% to 6.40%. However, the difference was statistically insignificant between A1B0 and A2B0 (P > 0.05). Compared to A0B0, co-application of swine manure biochar and biogas slurry increased the protein (43.45% ~ 100.29%), soluble sugar (15.96% ~ 104.74%), and starch (14.44% ~ 33.22%) contents of lotus root more significant. The difference was the most significant under the A2B2 treatment (P < 0.05).

**Table 2:** Effects of swine manure biochar and biogas slurry application on the quality of lotus root.

Treatment	Nitrate (mg·kg^-1^)	Protein (mg·g^-1^)	soluble sugar (mg·g^-1^)	starch (mg·g^-1^)
CK	112.1 ± 2.071h	0.153 ± 0.044f	2.480 ± 0.350e	6.512 ± 0.221b
A0B0	136.4 ± 2.692d	0.192 ± 0.011ef	2.638 ± 0.127de	6.629 ± 0.317b
A0B1	122.8 ± 2.045e	0.275 ± 0.015cd	3.038 ± 0.765de	7.152 ± 0.215ab
A0B2	145.3 ± 1.835c	0.320 ± 0.014bc	4.191 ± 0.772bc	7.901 ± 1.892ab
A1B0	113.9 ± 1.102gh	0.223 ± 0.016de	2.653 ± 0.356de	7.038 ± 1.373ab
A1B1	116.3 ± 2.112fg	0.276 ± 0.019cd	3.059 ± 0.086de	7.587 ± 0.665ab
A1B2	160.6 ± 0.761b	0.348 ± 0.030ab	4.980 ± 0.805ab	8.033 ± 0.456ab
A2B0	117.8 ± 2.966f	0.227 ± 0.029de	3.010 ± 0.469de	7.054 ± 0.107ab
A2B1	174.9 ± 1.974a	0.284 ± 0.042cd	3.606 ± 0.536cd	7.868 ± 0.456ab
A2B2	137.2 ± 1.326d	0.386 ± 0.070a	5.400 ± 0.838a	8.832 ± 1.201a

Different lowercase letters indicate significant differences between treatments (p < 0.05).

Nitrate contents increased in all treatments compared to CK. The A0B0 treatment significantly increased nitrate content of lotus root by 21.26% over CK. Compared to CK, swine manure biochar application increased the nitrate content of lotus root by 9.56% to 29.54%. 2% swine manure biochar application increased the nitrate content of lotus root by 6.52% compared to A0B0. However, 1% swine manure biochar application decreased the nitrate content of lotus root by 9.91% compared to A0B0. Biogas slurry application also increased the nitrate content of lotus root by 1.62% to 5.06% compared to CK. However, A1B0 and A2B0 were reduced by 16.44% and 13.61% compared to A0B0. In the presence of 1% swine manure biochar, the reapplication of biogas slurry increased the nitrate content of lotus root by 3.71% to 43.20% over CK. In the presence of 2% swine manure biochar, reapplication of biogas slurry increased the nitrate content of lotus root by 22.34% to 55.97% over CK. Under A1B1 treatment, lotus root had the lowest nitrate content of 116.3 mg·kg^-1^, which was 14.72% lower than A0B0.

In summary, swine manure biochar and biogas slurry application significantly increased the protein, soluble sugar, and starch contents of lotus root, with the effect most significant under A2B2 treatment (P < 0.05). In the presence of 1% swine manure biochar, the nitrate content of lotus root increased with increasing amount of biogas slurry application. In the presence of 2% swine manure biochar, the nitrate content of lotus root decreased with increasing amount of biogas slurry application.

#### 3.4.2 Effects of biochar and biogas slurry application on lotus root yield and nitrogen agronomic efficiency

**Figure 12:**
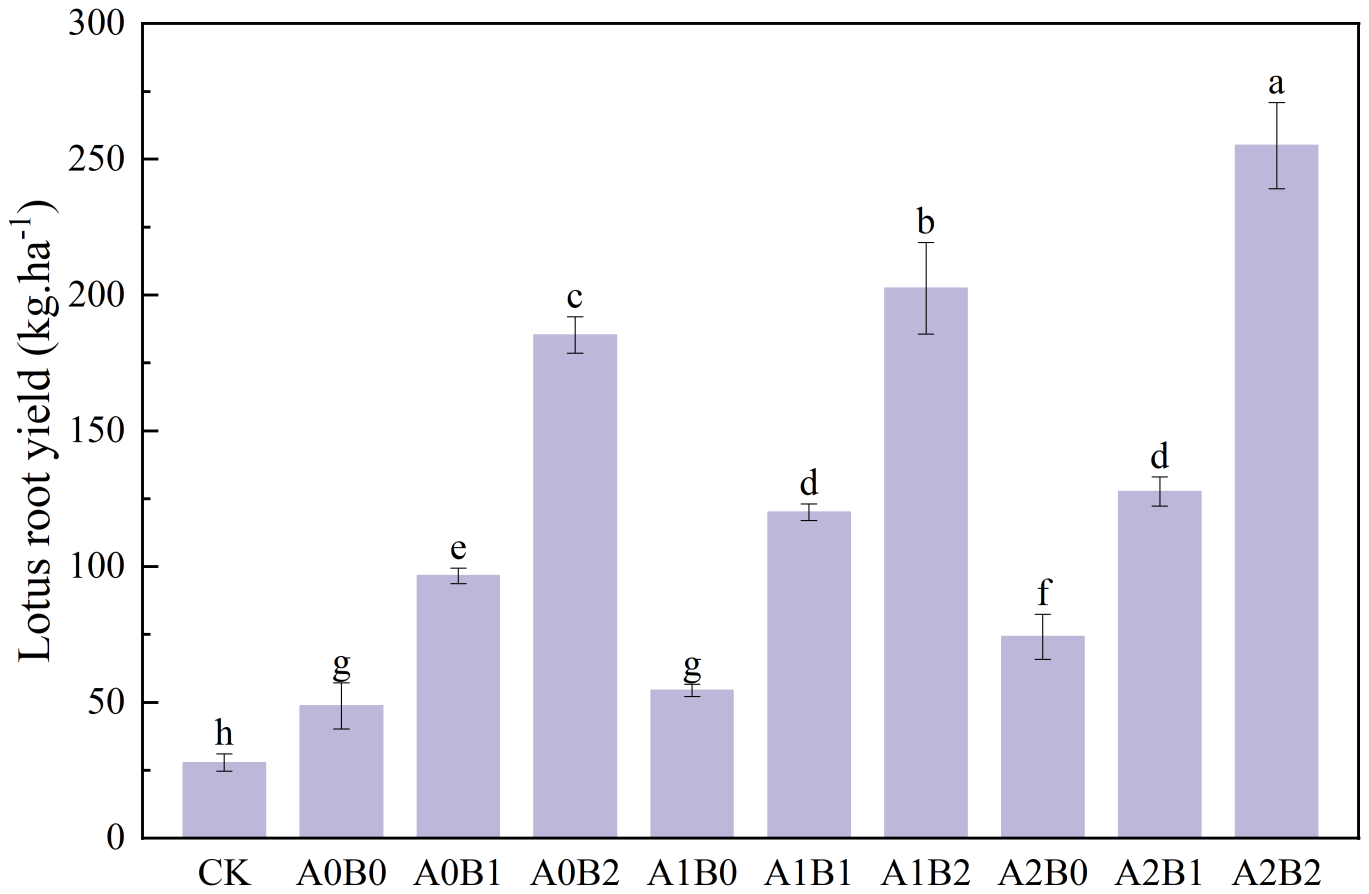
Effects of swine manure biochar and biogas slurry application on lotus root yield. Data over bars marked by different letters are significantly different at p < 0.05. Each value represents the mean of three replicates ± SE.

**Figure 13:**
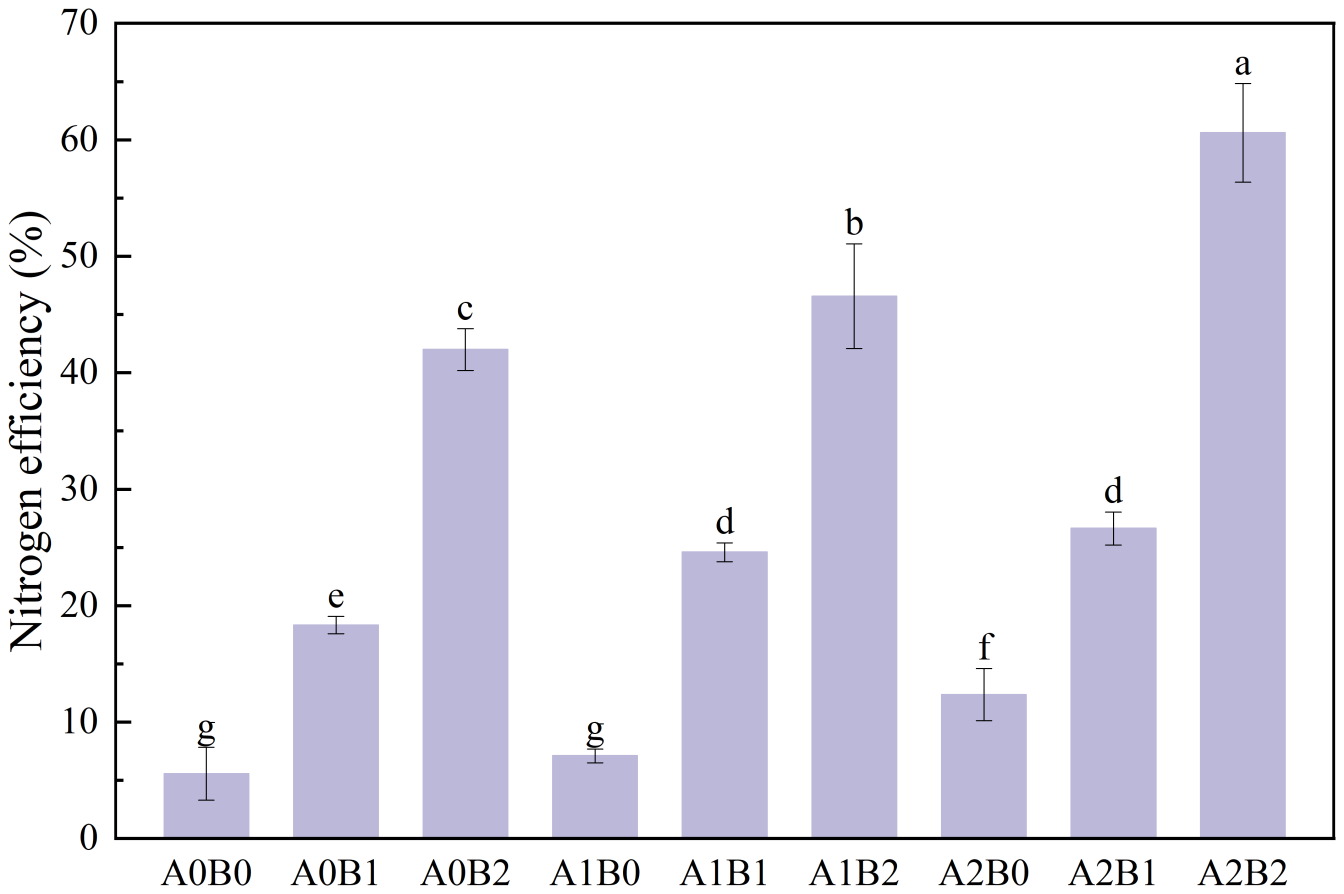
The nitrogen agronomic efficiency of different treatments. Data over bars marked by different letters are significantly different at p < 0.05. Each value represents the mean of three replicates ± SE.

The effects of swine manure biochar and biogas slurry application on lotus root yield are shown in **Figure 12**. Compared with CK, either the application of chemical fertilizer, swine manure biochar, biogas slurry or biochar-biogas combination could enhance lotus root yield. The A0B0 treatment enhanced lotus root yield by 74.96%, compared to CK. Compared with A0B0, single application of swine manure biochar and biogas slurry enhanced lotus root yield by 98.19% to 280.21% and 11.81% to 52.25%, respectively. The co-application of swine manure biochar and biogas slurry was better than the single application of both for lotus root yield enhancement. In the presence of 1% swine manure biochar, reapplication of biogas slurry increased lotus root yield by 146.27% to 315.45% over A0B0; in the presence of 2% swine manure biochar, reapplication of biogas slurry increased lotus root yield by 161.99% to 423.36% over A0B0. The nitrogen agronomic efficiency of different treatments is shown in **Figure 13**. Swine manure biochar application could significantly increase the nitrogen efficiency rate of lotus root (P < 0.05). The nitrogen utilization rate of lotus root of A0B2 was the highest, which was 6.54 times higher than A0B0. Biogas slurry application could also improve the nitrogen efficiency rate of lotus root, which is 0.28 times to 1.22 times higher than A0B0. Compared with A0B0, the application of swine manure biochar and biogas slurry significantly increased the nitrogen utilization rate of lotus root by 3.41times to 9.88 times (P < 0.05). Overall, swine manure biochar could still enhance lotus root yield after one year of application, with the best enhancement effect at 2% application rate. Swine manure biochar and biogas slurry application significantly enhanced lotus root yield, which reached a maximum value of 255.08 kg·ha^-1^ under A2B2 treatment. The nitrogen efficiency of lotus root reached the maximum of 60.59% under A2B2 treatment.

### 3.5 Correlation between lotus root quality and environmental factors

**Figure 14:**
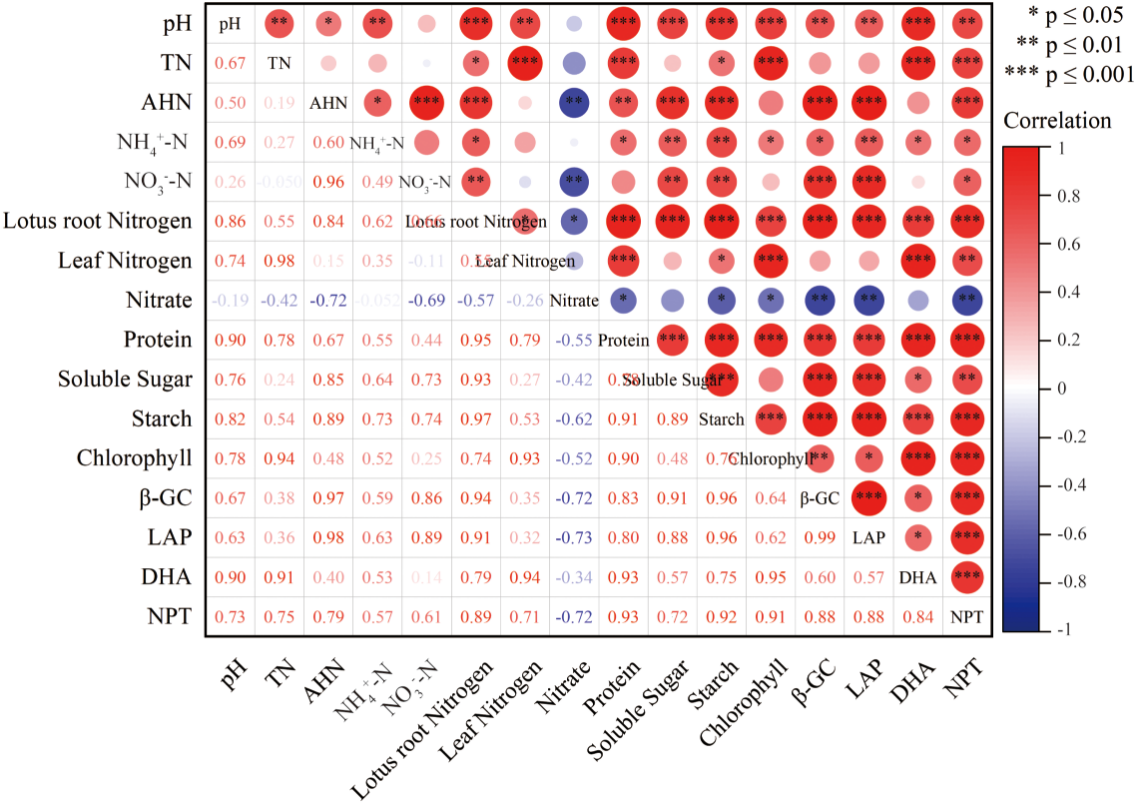
Correlation coefficient between lotus root quality and environmental factors. * statistically significant at the 0.05 level; ** statistically significant at the 0.01 level; *** statistically significant at the 0.001 level.

To specifically analyze the main factors that cause the change in the quality of lotus root in different treatments, this paper selected twelve environmental factor indicators of pH, TN, AHN, NH_4_^+^-N, NO_3^-^_-N, lotus root nitrogen, leaf nitrogen, leaf chlorophyll, β-GC, LAP, DHA and NPT, to analyze the correlation of changes in environmental factors to changes in lotus root quality. The results are shown in **Figure 14**. From the overall results, the changes of lotus root quality are closely related to environmental factors. Lotus root nitrate content was significantly negatively correlated with AHN, NO_3_^-^-N, β-GC, LAP, NPT and lotus root nitrogen content. Lotus root protein content was significantly positively correlated with pH, TN, AHN, DHA and NPT contents. Lotus root soluble sugar content was significantly positively correlated with AHN, β-GC and LAP contents. Lotus root starch content was significantly positively correlated with pH, AHN and β-GC content. Soil AHN content was significantly positively correlated with NO_3_^-^-N and lotus root nitrogen content and significantly negatively correlated with lotus root nitrate content.

**Figure 15:**
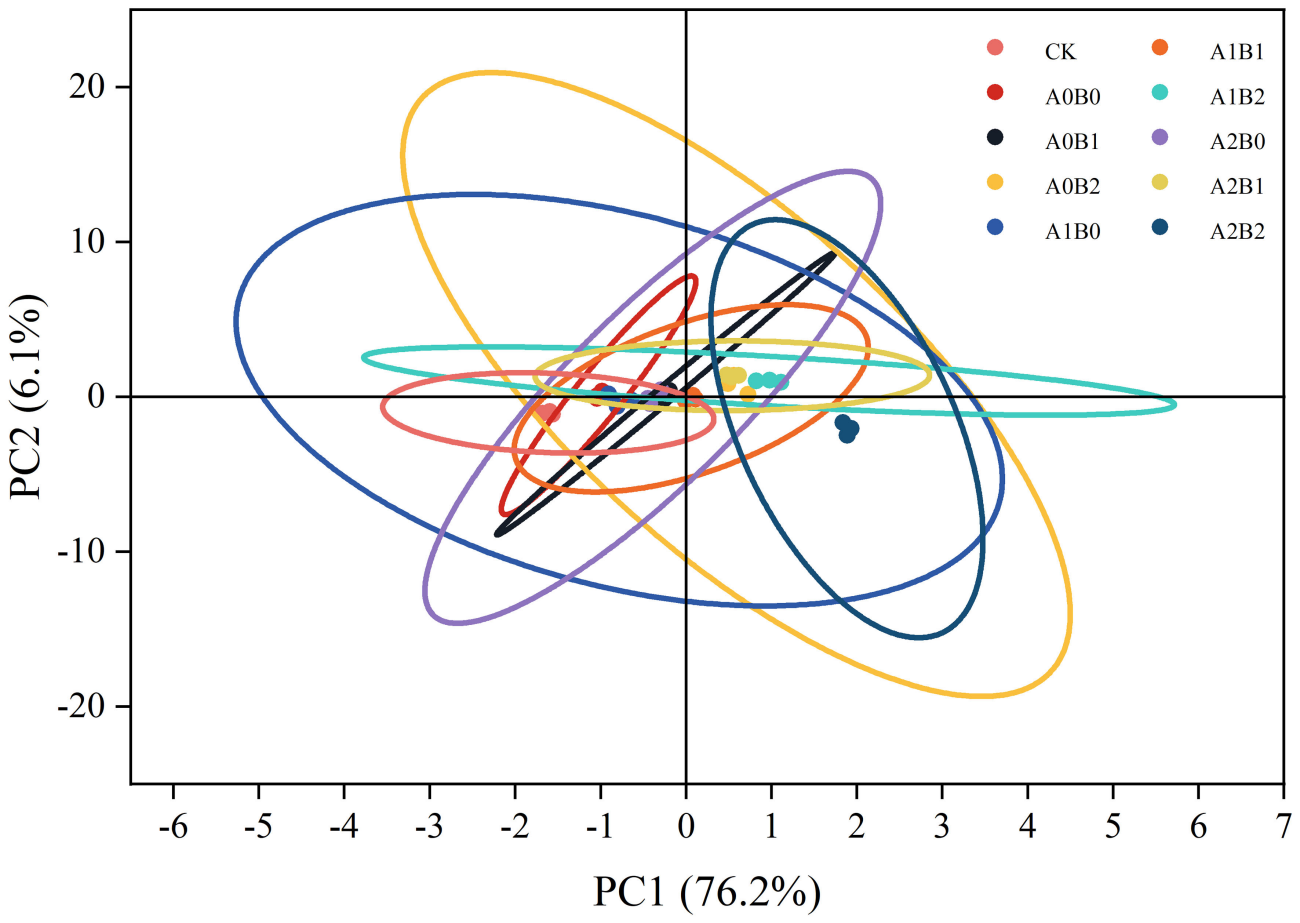
The principal component analysis.

The data was subjected to principal component analysis and the results are shown in **Figure 15**. The variance contribution rates of the first and second principal components were 76.2% and 6.1%, respectively, which can well explain the correlation and variability among different treatments, thus realizing the differentiation of different treatments. The difference between A1B0 and A2B0 treatments was not significant, while the differences between CK, A0B0, A0B1, A0B2, A1B1, A1B2, A2B1 and A2B2 treatments were significant.

## 4 Discussion

### 4.1 Effects of biochar and biogas slurry application on soil nitrogen content

Biochar with porous structure and large specific surface area can significantly improve soil aeration, enhance nitrogen adsorption and reduce nitrogen leaching (Xu et al., 2021), improving nitrogen utilization effectively (Zhang et al., 2021). Biochar application significantly increased TN, and NH_4_^+^-N in paddy soil and provided more nitrogen sources for microorganisms. The enhancement intensity increased with the increasing biochar application (Yang et al., 2019). In this study, after one year of swine manure biochar application, the soil TN, NH_4_^+^-N and NO_3_^-^-N contents were still significantly higher than A0B0, consistent with previous research (Yu et al., 2020) in aquatic vegetable watercress origin. Chen et al (Chen et al., 2015) showed that the soil TN and mineral nitrogen (NH_4_^+^-N and NO_3_^–^N) contents of paddy fields increased after biogas slurry watering. Song et al (Song et al., 2023) concluded that biogas slurry application significantly affected soil nitrogen content in the integrative analysis of the effect of biogas slurry agriculture on soil nitrogen patterns. A similar trend was observed in the present study, where the biogas slurry application significantly increased soil nitrogen content.

Biochar and biogas slurry application can reduce nutrient leaching from biogas slurry and soil, thus improving the utilization of biogas slurry and enhancing soil nutrient content (Wang et al., 2018). In the study of the effect of straw biochar combined with biogas slurry on soil organic matter and total nitrogen content, Zheng et al (Zheng et al., 2020a) concluded that the surface functional group adsorption of biochar and the slow release of nitrogen from biogas slurry under the action of biochar made the application of biochar and biogas slurry played a positive role in increasing soil total nitrogen content. Adding swine manure biochar on top of biogas slurry application could further increase soil total and microbial nitrogen content (Singla et al., 2014). In this study, swine manure biochar still showed absorption and fixation of nitrogen in biogas slurry and fertilizer after one year of application, thus significantly enhancing soil nitrogen content, which agrees with their research. The soil nitrogen content reached its highest value at the rooting period with the prolongation of lotus root fertility, which may be due to the following reasons: (1) Swine manure biochar with large specific surface area and positively charged characteristics facilitates the transformation and uptake of nitrogen in the soil and reduces the leaching loss of nitrogen, thus increasing the soil soluble nitrogen (SN) content. (2) The rich microporous structure of swine manure biochar provides attachment points for microbial growth and reproduction, which is conducive to improving soil microbial activity, thus promoting microbial degradation of organic nitrogen in soil and releasing ammonium nitrogen and nitrate nitrogen. (3) Because biogas slurry is rich in inorganic salt and reactive organic nitrogen, which can increase soil mineral nitrogen (NH_4_^+^-N and NO_3_^-^-N) content.

### 4.2 Effect of biochar and biogas slurry application on soil enzyme activity

Soil enzymes are mainly from the secretions of plants, animals, and microbial cells. Soil enzymes are one of the most active components in the soil. They play an important role in regulating the decomposition of soil organic matter and nutrient cycling processes (Pan et al., 2021). Biochar application could significantly increase the activities of relevant enzymes involved in soil carbon and nitrogen cycles (Zhang et al., 2019). In this study, Swine manure biochar application significantly increased the activities of soil protease, leucine aminopeptidase, β-glucosidase, and dehydrogenase after one year of application, consistent with previous research. Rice straw biochar application in 30,000 kg·hm^-2^ significantly increased β-1,4-glucosidase, β-1,4-N-acetylglucosaminidase, and leucine aminopeptidase activities by 23.1% to 47.9%, which indicated that biochar application promoted soil biochemical reaction, accelerated soil carbon and nitrogen cycling, thus improving the availability of soil nutrients (Lu et al., 2022). Xu et al (Xu et al., 2019) used a field location test to study the holding effect of rice straw biochar one-time application for six years on soil fertility and enzyme activity in paddy fields, which obtained that biochar still significantly enhanced soil dehydrogenase activity by 27.5% after six years of application.

In this study, biogas slurry application also increased soil protease and dehydrogenase activities, which is consistent with the conclusion of Wu et al (Wu et al., 2021), which is biogas slurry long-term application significantly increased soil protease activity by 9.20% to 44.69% and dehydrogenase activity by 4.94% to 47.44%. In this study, compared to CK and A0B0, the co-application of swine manure biochar and biogas slurry significantly enhanced soil enzyme activity, which is in agreement with the results of Wang et al (Wang, 2023), which is the co-application of swine manure biochar and biogas slurry further intensified soil enzyme activity, emphasizing their positive synergistic impact.

### 4.3 Effects of biochar and biogas slurry application on nitrogen content and quality of lotus root

Biochar application in soil has multiple benefits, including regulating soil pH, promoting nutrient element effectiveness, and improving water-fertilizer-retention properties. These effects subsequently contribute to the growth of crops and the increase in yield (Jindo et al., 2020; Enaime and Lübken, 2021). In the current study, swine manure biochar exhibited a sustained and significant increase in the nitrogen content of lotus root after one year of application. This finding indicated that swine manure biochar application is beneficial to the uptake and accumulation of nitrogen in the crop, consistent with previous studies’ findings. Duan et al (Duan et al., 2017) showed that wheat straw biochar application led to a higher accumulation of nitrogen, potassium nutrients, and dry matter of the aboveground part in a coupled experiment of biochar and watercress wetland system. Qu et al (Qu et al., 2012) also verified this positive impact, who studied the effect of wheat straw biochar on rice yield and nitrogen utilization in late-season rice. Applying biochar has been shown to enhance nitrogen uptake in crops, increasing the amount of dry matter in the aboveground portion. Biochar application significantly elevated SPAD value of rice leaves at whole growth periods (Sui et al., 2018), thus enhancing rice yield.

Biogas slurry application also demonstrated positive effects on crop yield and quality. Zhao et al (Zhao et al., 2014) found that substituting chemical fertilizer nitrogen with 0 to 75% biogas slurry effectively promoted watercress growth, increased watercress yield and appropriately improved watercress quality. The study indicated that at the ratio of biogas slurry replacing chemical fertilizer is appropriate at about 50%. Similarly, Wang et al (Wang et al., 2004) reported an average increase of 812.7 kg/pond in lotus root yield after biogas slurry application during lotus root cultivation. In this study, biogas slurry application not only increased the protein, soluble sugar and starch content of lotus roots but also enhanced yield. These findings align with existing results. Interestingly, the co-application of swine manure biochar and biogas slurry demonstrated a synergistic effect on the nitrogen content and quality of lotus root. Biochar and biogas slurry, as commonly used soil amendments, can improve soil properties and affect the migration of soil nutrients. However, the effect of their application on the properties of flooded soils has rarely been studied. In this manuscript, we did novel research to investigate their effect in lotus cultivation with a pot experiment.

## 5 Conclusions

Swine manure biochar still positively affected soil nitrogen content, enzyme activity, and lotus root quality after one year of application. During the rooting period (18 months after biochar application), swine manure biochar application increased soil nitrogen content by 2.22% to 64.71% compared with A0B0. The co-application of swine manure biochar and biogas slurry significantly increased soil nitrogen contents, and the A2B2 treatment was the most significant enhancement effect during whole fertility periods of lotus root. The activities of soil protease, leucine aminopeptidase, β-glucosidase, and dehydrogenase tended to increase and decrease with the prolongation of lotus root fertility periods, reaching their peak values during the rooting period. Soil enzyme activities were most significantly elevated under 2% swine manure biochar application. Lotus roots protein, soluble sugar and starch contents were maximized under A2B2 treatment. Nitrate contents decreased with the increase of biogas slurry application under 2% swine manure biochar application.

## Data availability statement

The raw data supporting the conclusions of this article will be made available by the authors, without undue reservation.

## Author contributions

MZ: Writing – original draft. JJ: Writing – original draft. MM: Methodology, Writing – original draft. ZJ: Writing – review & editing. MW: Writing – review & editing. SS: Funding acquisition, Writing – review & editing. LP: Writing – review & editing.
